# Maxillary protraction using a hybrid hyrax-facemask combination

**DOI:** 10.1186/2196-1042-14-5

**Published:** 2013-05-20

**Authors:** Manuel Nienkemper, Benedict Wilmes, Alexander Pauls, Dieter Drescher

**Affiliations:** 1 University of Düsseldorf, Department of orthodontics, Moorenstr. 5, 40225 Düsseldorf, Germany

## Abstract

**Background:**

The aim of this in study was the evaluation of treatment outcomes after using a hybrid hyrax-facemask combination in growing class III patients.

**Methods:**

Treatment of 16 children (mean age 9.5 ± 1.3 years) was investigated clinically and by means of pre- and post-treatment cephalograms. Changes in sagittal and vertical, and dental and skeletal values were evaluated and tested for statistically significant differences.

**Results:**

All mini-implants remained stable during treatment. Mean treatment duration was 5.8 ± 1.7 months. There was a significant improvement in skeletal sagittal values: SNA, +2.0°; SNB, -1.2°; ANB, +3.2°; WITS appraisal, +4.1 mm and overjet, +2.7 mm. No significant changes were found concerning vertical skeletal relationships and upper incisor inclination. In relation to A point, the upper first molars moved mesially about 0.4 mm (*P* = 0.134).

**Conclusions:**

The hybrid hyrax-facemask combination seems to be effective for orthopaedic treatment in growing class III patients. Unwanted maxillary dental movements can be avoided due to stable skeletal anchorage.

## Background

Treatment of skeletal class III malocclusion still seems to be one of the most ambitious challenges in orthodontics. This kind of malocclusion can be caused by a retrognathic maxilla, a prognathic mandible or a combination of both [1]. A surgical correction after the completion of growth is unavoidable in many cases, especially in cases with a prognathic mandible.

For patients with maxillary deficiency, the use of a facemask for protraction of the maxilla is one of the most common therapies. It was introduced by Delaire in 1971 [2]. The orthopaedic treatment of class III malocclusion is particularly efficient in patients during the early developmental phases [3-7]. For this reason, treatment should start in the early mixed dentition. The literature provides evidence that this is an effective method to treat a maxillary deficiency [4].

The use of a facemask for class III correction may also cause problems. The forces for maxillary protraction are normally applied to the upper teeth. As a result, a significant mesial migration of the upper teeth can be observed [8]. This may cause severe anterior crowding and reduce the orthopaedic treatment effects [9].

To avoid this side effect, different kinds of anchorage protocols were described in the literature. First, artificially ankylosed teeth were used to reduce dental effects [10]. Later, dental implants and surgical plates transferred the forces directly to the upper jaw [11,12].

To increase the skeletal effect on the maxilla, facemask therapy is often combined with rapid maxillary expansion (RME). A stimulating effect on the midfacial sutures caused by distraction with an improved response on protraction is expected. Even though it was discussed controversially [13], the analysis of the literature data affirms the benefit of the treatment combination [4]. Because of the well-known problems caused by tooth-borne expansion devices such as buccal tipping, gingival recessions or root damage, techniques based on bone-borne devices are described. Pure bone-borne Rapid Palatal Expansion (RPE) device can be used [14,15]. Besides the high invasiveness for insertion, they may also cause root lesions and infections [14]. To minimize the invasiveness, Wilmes et al. have introduced the hybrid hyrax, a tooth- and bone-borne expander [16]. This device is connected to two orthodontic mini-implants in the anterior palate and is also attached to the first molars. Recently, it has been shown that the mentioned side effects of RME regarding the transverse direction can be minimised using a hybrid hyrax [16]. As another approach, it can be used for the treatment of class III malocclusion with maxillary expansion and protraction [16,17]. The aim of this study was to evaluate the treatment effects produced by the hybrid hyrax-facemask combination in growing class III patients.

## Methods

Inclusion criteria for this study were a mild to severe skeletal class III malocclusion (WITS appraisal ≤ 2.0 mm) and an age of up to 12 years. A sample of 16 patients (10 males, 6 females, mean age of 9.5 ± 1.3 years) treated with RME with hybrid hyrax and maxillary protraction with facemask was evaluated. This study was approved by the ethics committee of the University of Düsseldorf.

### Treatment protocol

**Figure 1:**
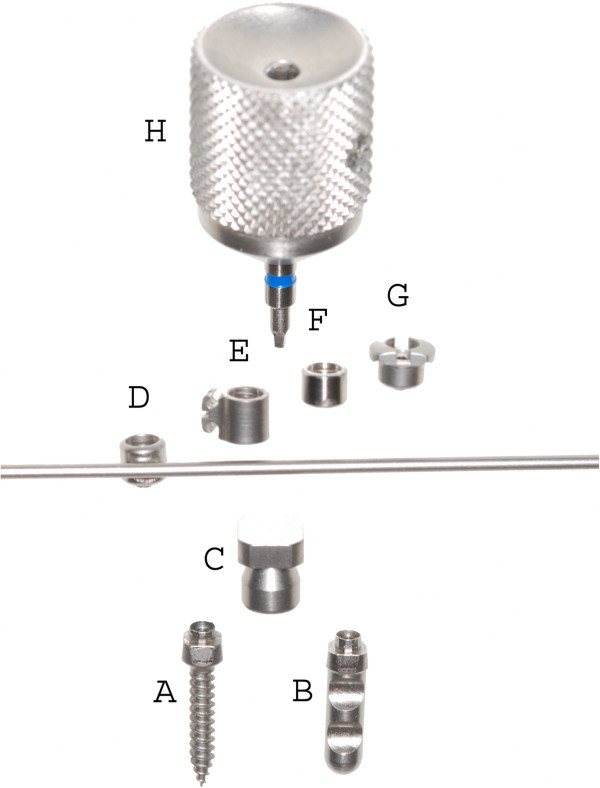
**Benefit-System (PSM Medical Solutions, Tuttlingen, Germany) with various abutments. A**, mini-implant; **B**, laboratory analogue; **C**, impression cap; **D**, wire abutment with wire in place; **E**, bracket abutment; **F**, standard abutment; **G**, slot abutment and **H**, screwdriver for abutment fixation.

**Figure 2:**
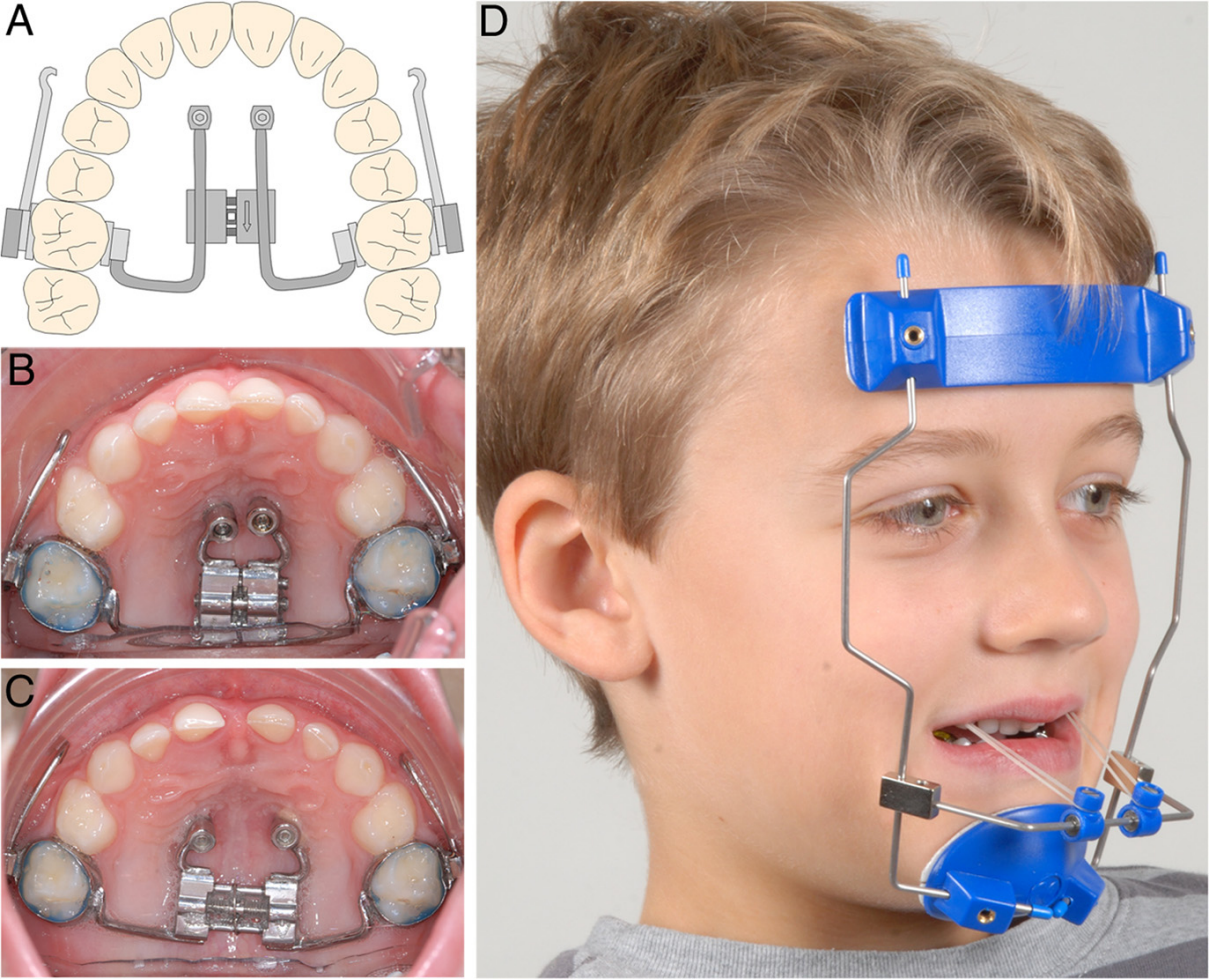
**The hybrid hyrax. **(**A**) Sketch of the modified hybrid hyrax device with rigid sectional wire for maxillary protraction *in situ*. (**B**) Hybrid Hyrax device *in situ*; because of the retarded dentition, the molar bands were fitted to the second deciduous molars. (**C**) Situation after rapid maxillary expansion (duration, 8 days). (**D**) Maxillary protraction with facemask and elastics; anterior-caudally angulated force direction of 20° to 30° with respect to occlusal plane.

**Figure 3:**
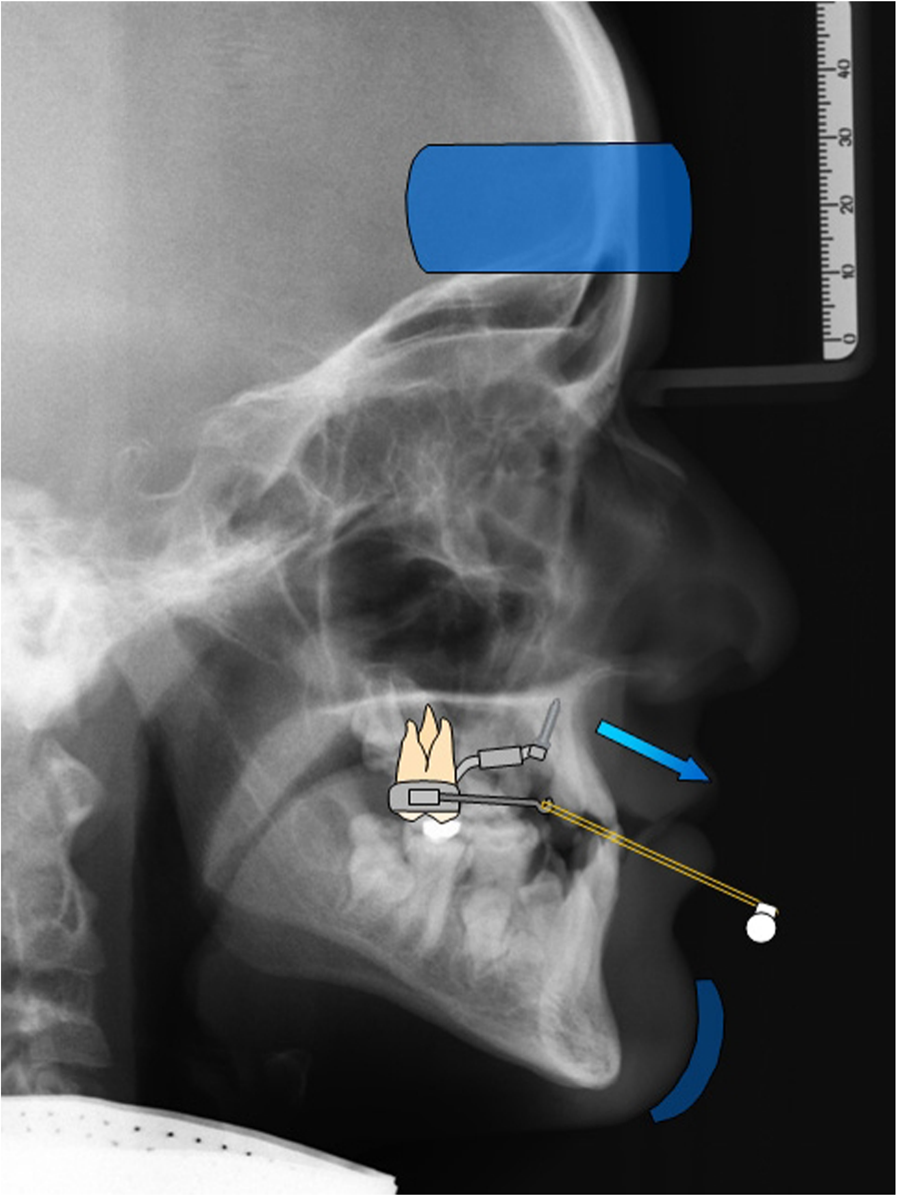
Biomechanical sketch of maxillary protraction using hybrid hyrax-facemask combination.

The first step was the insertion of two mini-implants in the anterior palate on both sides of the midpalatal suture. After local anaesthesia, the soft tissue thickness was measured using a dental probe. Insertion in a region with thin mucosa is very important regarding the biomechanical loading capacity. Treating only young patients with this protocol, pre-drilling was not needed. Benefit mini-implants (PSM Medical Solutions, Tuttlingen, Germany) (Figure 1) of size 2 × 9 mm can be inserted directly. They should be angled approximately parallel to each other. Orthodontic bands were fitted to the first molars, and transfer caps (B in Figure 1) were adapted to the implants' head. For precise transfer of the implants' position, the caps were connected using light-curing composite. Afterwards, a silicon impression was taken in which the transfer caps and molar bands were placed. The laboratory analogues (C in Figure 1) were inserted into the impression caps, and a plaster cast was made. After curing, two standard abutments (F in Figure 1) were screwed to the laboratory analogues. A stainless steel wire 1.5 mm in diameter was used to connect a molar band, a split palatal screw (Hyrax, Dentaurum, Ispringen, Germany) and an abutment on each side by welding. For application of orthopaedic protraction forces, the hybrid hyrax was modified by welding rigid sectional wires (stainless steel, diameter 1.2 mm) featuring a hook to the buccal side of the molar bands (Figure 2A). The hooks were positioned at the canine region to enable a line of force anterior to the centre of resistance of the maxilla (Figure 3).

During the next appointment, the modified hybrid hyrax was inserted by screwing on the abutments and fitting the molar bands. The bands were fixed by light-curing glass ionomer cement allowing adequate time for application. RME was performed by activating the split screw by 90° turns four times a day, which means a daily expansion of 0.8 mm (Figure 2B,C). A transversal overcorrection of 30% was achieved for relapse compensation. In cases with little maxillary transversal deficiency, expansion was performed anyway for stimulation of the midfacial sutures. The split screw was then ‘deactivated’ in the opposite direction afterwards.

**Figure 4:**
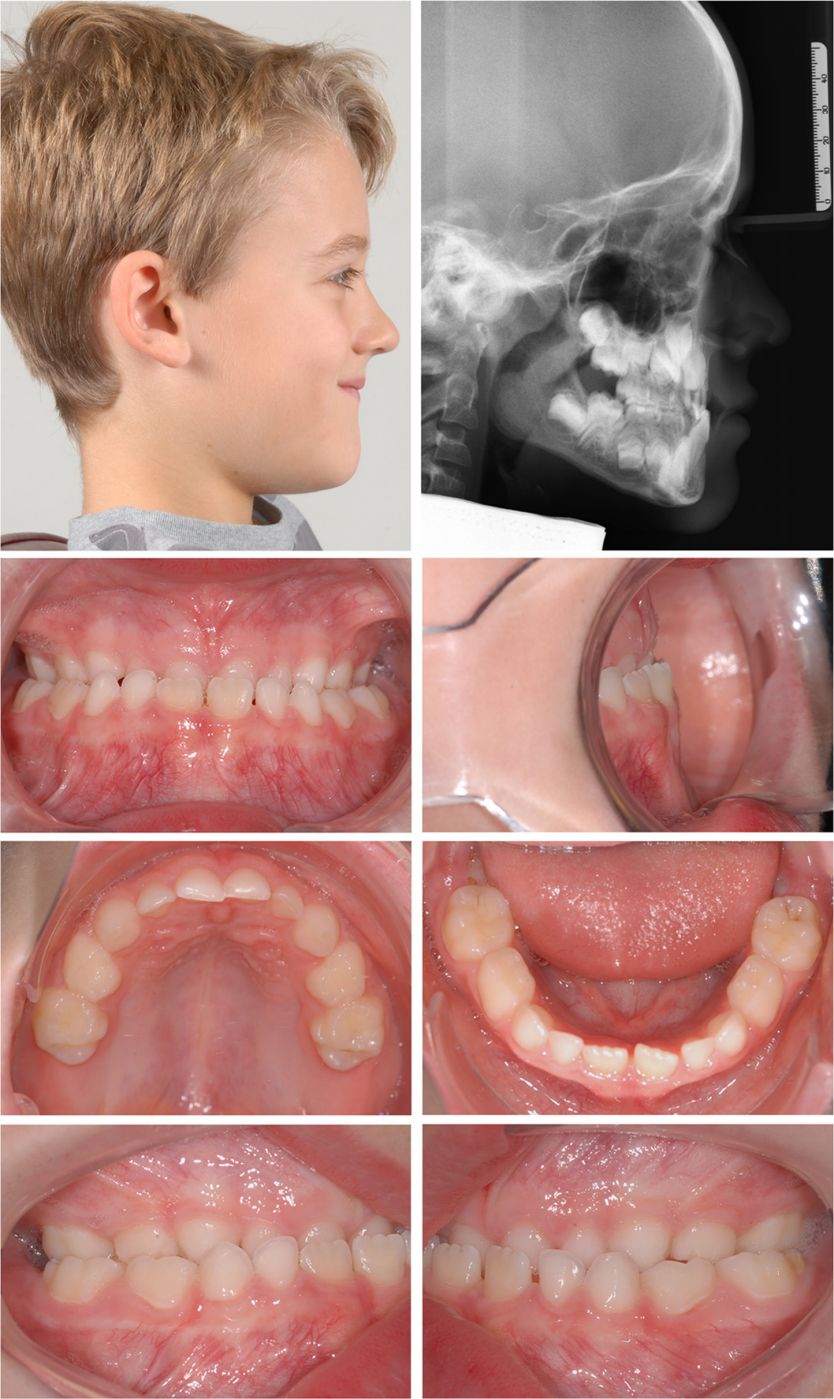
An 8-year-old male patient with severe skeletal and dentoalveolar class III malocclusion before treatment.

**Figure 5:**
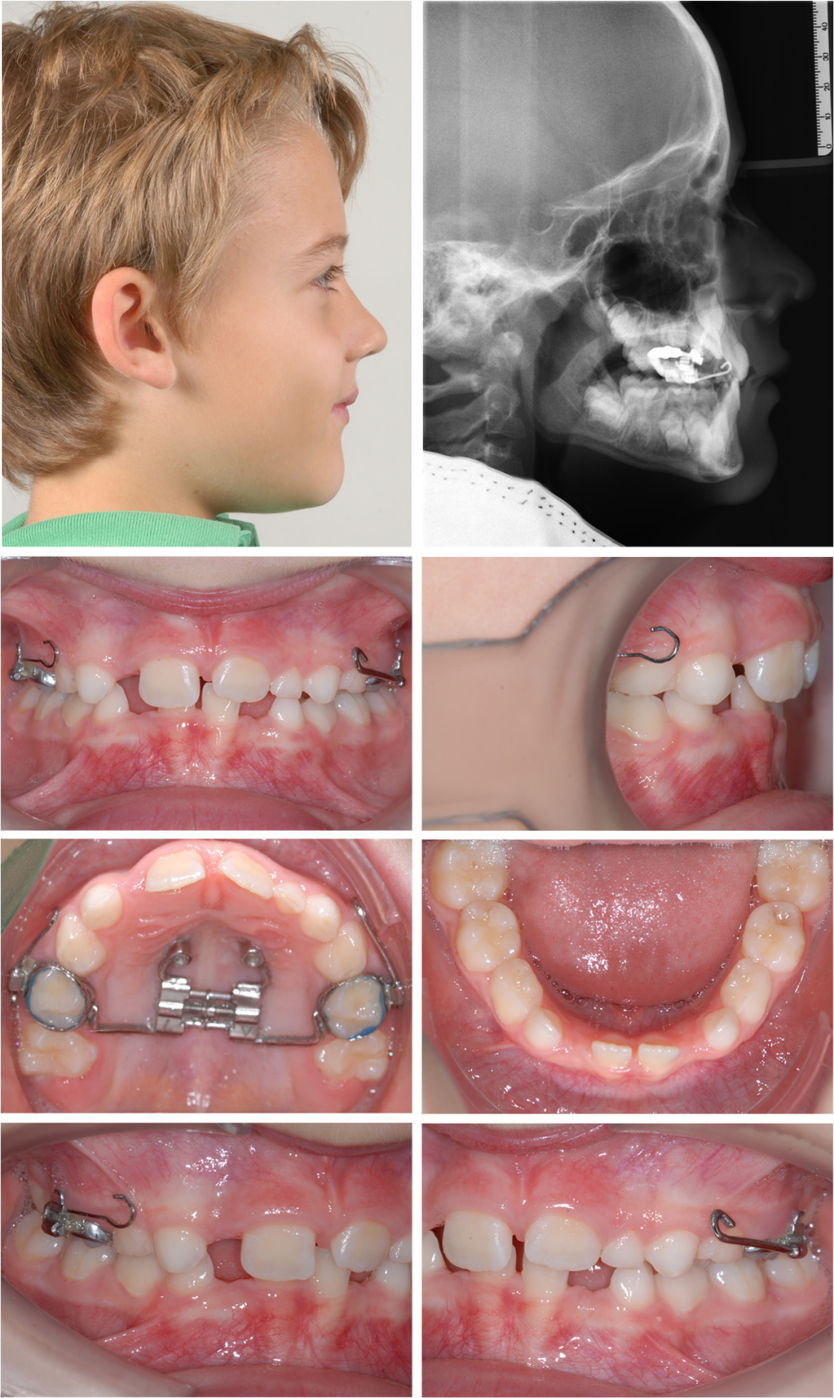
Situation after 10 months of treatment.

Maxillary protraction was started simultaneously with the screw activation. The facemask was adjusted in order to apply an anteriorly and caudally angulated force with an inclination of 20° to 30° to the occlusal plane (Figure 2D). A force of 400*g* was applied on each side by elastics. The amount of force was clinically controlled using a force gauge. As a clinical example, the treatment of an 8-year-old male patient is shown (Figures 2, 4, 5; Table 1).

**Table 1:** Comparison of cephalometric changes in the 8-year-old male patient before (T1) and after (T2) treatment

	**T1**	**T2**	**T2-T1**
**Angular measurements (deg)**			
**SNA**	82.5	84.7	2.2
**SNB**	84.2	80.7	-3.5
**ANB**	-1.8	4.0	5.8
**ML-NL**	21.7	23.9	2.2
**U1-NL**	85.6	94.7	9.1
**Linear measurements (mm)**			
**WITS**	-5.1	1.5	6.6
**Overbite**	3.1	1.5	-1.6
**Overjet**	-1.9	2.5	4.4
**U6-A point**	28.5	28.3	-0.2

### Evaluation of treatment outcomes

**Figure 6:**
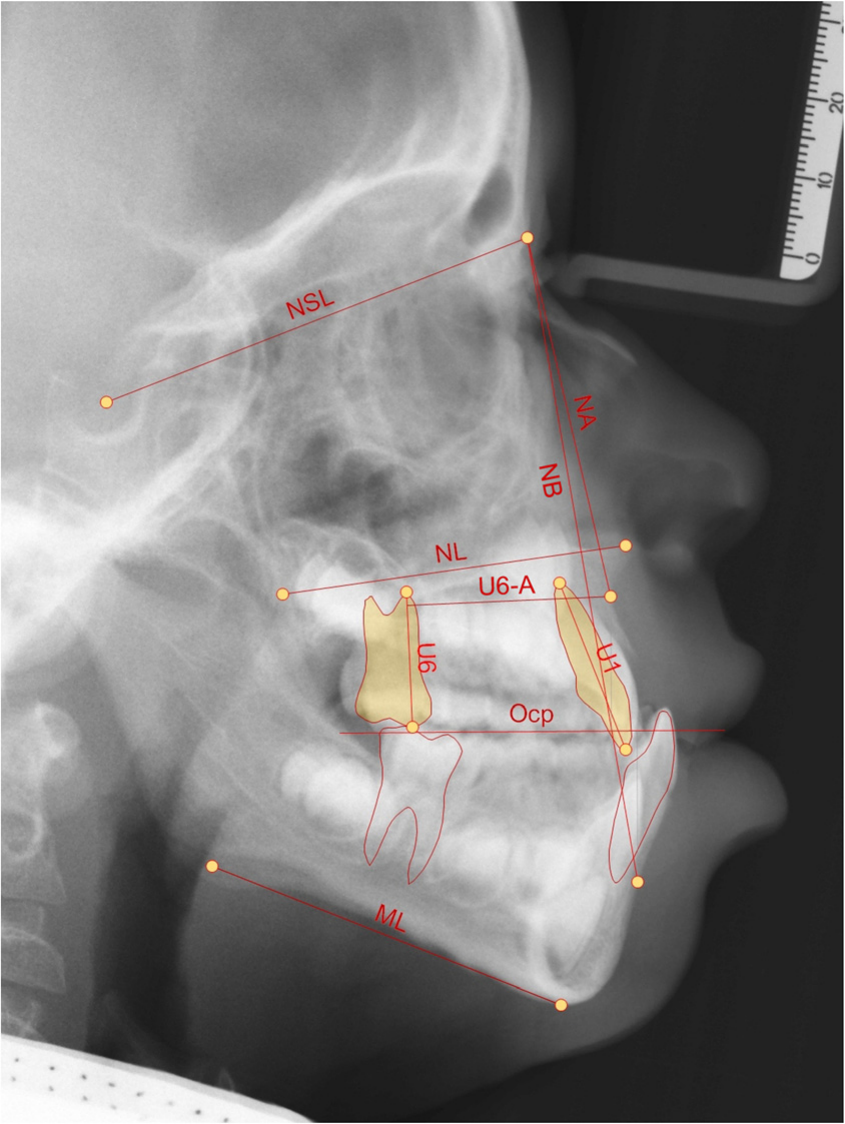
Cephalometric points, vertical and sagittal measurements used.

**Figure 7:**
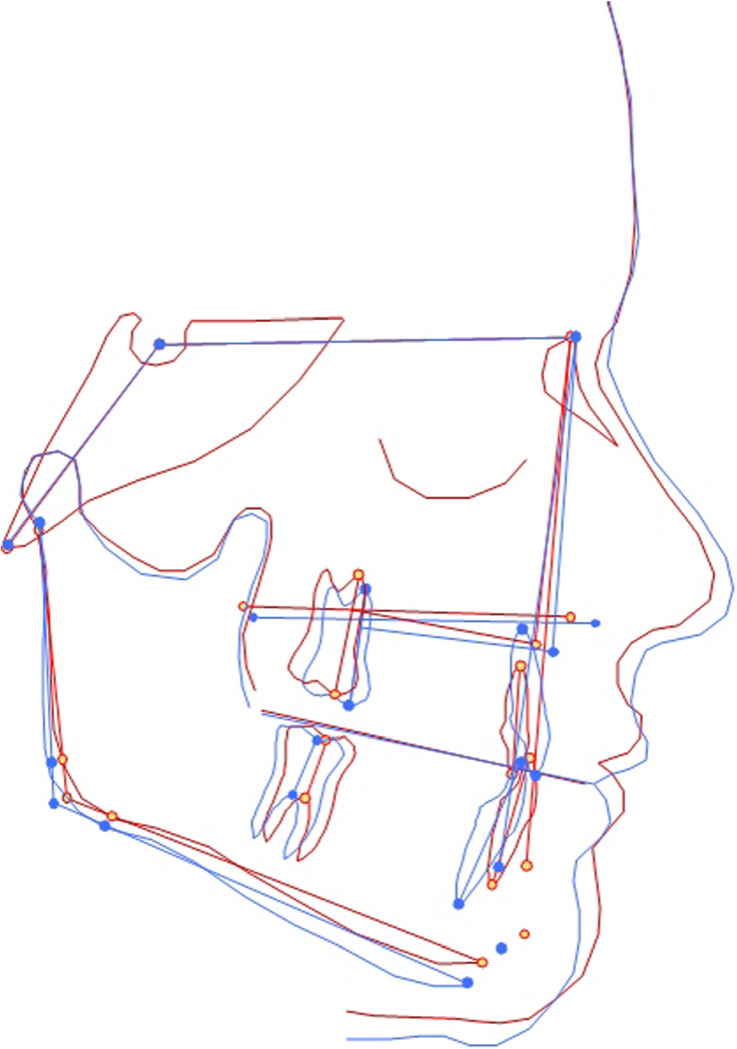
Superimposition of pre-(red) and post-treatment (blue) cephalograms of the 8-year-old male patient.

Changes in SNA and SNB angles, WITS appraisal and overjet were analysed to assess sagittal improvement (Figures 6 and 7). ML-NL angle and overbite changes were observed to document vertical effects. Upper incisor inclination changes and changes in the distance between the upper first molar and A point were investigated to identify maxillary tooth movements. All values were tested for normal distribution by the Shapiro-Wilk test. Pre- and post-treatment differences were tested for statistical significance using paired *t* test. Only differences in the distance between the upper first molars and A point were tested by Wilcoxon test since the respective data sets did not show normal distribution. The levels of significance used were *P* < 0.05 and *P* < 0.001. All statistics were performed using SPSS version 19 (IBM, Armonk, NY, USA).

### Method error

Cephalograms were taken using digital X-ray. Measurements and superimpositions were performed by the same operator and verified by a second operator.

For determination of the method error, ten randomly selected cephalograms were measured again within a week by the same operator. Random errors according to Dahlberg [18] and coefficients of reliability [19] were calculated.

Random error ranged from 0.11 to 0.41 mm for linear measurements and from 0.19° to 0.60° for angular measurements. The coefficient of reliability ranged from 0.90 to 0.99 for linear measurements and from 0.95 to 0.99 for angular measurements.

## Results

Mean treatment duration was 5.8 ± 1.7 month. All mini-implants showed high primary stability and remained stable during treatment. There was a highly significant (*P* < 0.001) increase in SNA (+2.0°) and ANB (+3.2°) (Table 2). SNB decreased significantly by -1.2° (*P* < 0.05). Highly significant improvements of WITS appraisal (+4.1 mm, *P* < 0.001) and overjet (+2.7 mm, *P* < 0.001) were found. There were no significant differences in the vertical dimension regarding ML-NL angle or overbite changes. There was no significant maxillary tooth movement regarding upper incisor angle and distance between the upper first molar and A point. In relation to the A point, the upper first molars moved mesially about 0.4 mm (*P* = 0.134).

**Table 2:** Comparison of cephalometric changes before (T1) and after treatment (T2)

	**T1**		**T2**		**T2-T1**		***P *** **value**
	**Mean**	**SD**	**Mean**	**SD**	**Mean**	**SD**	
**Angular measurements (deg)**							
**SNA**	79.8	4.8	81.8	5.0	2.0	2.0	<0.001^a^
**SNB**	80.7	4.2	79.5	4.7	-1.2	2.3	<0.05^a^
**ANB**	-0.9	2.3	2.3	3.2	3.2	1.9	<0.001^a^
**ML-NL**	27.3	5.3	28.0	7.1	0.7	3.3	0.440^a^
**U1-NL**	107.5	10.6	107.3	7.8	-0.2	7.9	0.911^a^
**Linear measurements (mm)**							
**WITS**	-4.8	2.1	-0.7	2.2	4.1	2.1	<0.001^a^
**Overbite**	0.3	2.6	0.1	1.4	-0.2	2.2	0.783^a^
**Overjet**	-0.2	2.2	2.5	1.5	2.7	2.5	<0.001^a^
**U6-A point**	24.4	2.6	24.0	2.7	-0.4	1.0	0.134^b^

^a^Paired *t* test; ^b^Wilcoxon test.

## Discussion

The hybrid hyrax-facemask combination was designed to improve orthopaedic treatment of class III malocclusion in growing patients. Side effects such as maxillary tooth movement should be avoided by employing skeletal anchorage. The effectiveness of hybrid hyrax appliances regarding RME has already been demosntrated [16].

Significant sagittal skeletal improvement could be achieved as shown by changes in SNA and WITS appraisal. A meta-analysis of treatment effects achieved by conventional RME and facemask revealed a SNA improvement by 1.4° [4]. The result of the current investigation suggests a higher effectiveness regarding maxillary anterior advancement. Using rigid buccal sectional wires with hooks and anterior-caudal force direction, vertical side effects such as bite opening could be avoided.

Tooth movement is one of the major problems in performing maxillary protraction using a tooth-borne RME device [9,20]. In addition to greater skeletal effects, maxillary tooth movement could be inhibited using hybrid hyrax devices.

Skeletal effects would have been even greater if patients were treated at a younger age (mean age, 9.5 ± 1.3 years). Maxillary protraction is more effective if it is started before the age of 8 years [4]. In older patients with reduced skeletal response, there is a high risk of dental side effects. Contrary to conventional RPE device, there is no need of anterior tooth anchorage using the hybrid hyrax device. This is advantageous in patients whose deciduous teeth already show advanced root resorption or are missing.

The mini-implants showed high primary stability and remained stable during treatment. One explanation for this high success rate might be the fact that the implants were inserted in the anterior palate which provides very good bone quality. Another advantage of the insertion region is the fact that root contact or traumatic interference with anatomical structures is rather unlikely [21,22]. The abundant space available enabled us to insert implants with larger diameters which also improve implant stability [23,24]. The stable screw coupling to the appliance avoids tipping of the mini-implants which leads to an increased biomechanical load capacity. Thus, skeletal anchorage remains stable during RPE and maxillary protraction using orthopaedic forces. Using only two mini-implants for skeletal anchorage, insertion of a hybrid hyrax appears to be minimally invasive compared to skeletal anchored transpalatal distractors based on surgical plates.

## Conclusions

The hybrid hyrax-facemask combination seems to be effective for orthopaedic treatment in growing class III patients. Significant sagittal improvement of the maxilla and inhibition of the mandible can be achieved. Unwanted maxillary dental movements can be avoided due to stable skeletal anchorage. The surgical invasiveness is comparatively low.

## Consent

The authors state, that the consent for using the photos was obtained from the child’s parents.

## Competing interests

Dr. Wilmes is the inventor of the Benefit-System.

## Authors’ contributions

MN carried out the measurements and statistical analysis and drafted the manusscript. AP and DD took part in performing the superimpositions. BW and DD took part in writing the paper.
